# Limit Laws for Sums of Logarithms of *k*-Spacings

**DOI:** 10.3390/e27040411

**Published:** 2025-04-10

**Authors:** Paul Deheuvels

**Affiliations:** Laboratoire de Statistique Théorique et Appliquée, Université Paris VI, 7 Avenue du Château, F 92340 Bourg-la-Reine, France; pd@ccr.jussieu.fr

**Keywords:** order statistics, parametric hypothesis testing, hypergeometric functions

## Abstract

Let $Z=Z_{1},\ldots ,Z_{n}$ be an i.i.d. sample from the absolutely continuous distribution function F(z):=P(Z≤z), with density $f\left(z\right):=\frac{d}{dz}F\left(z\right)$. Let $Z_{1,n}<\ldots <Z_{n,n}$ be the order statistics generated by $Z_{1},\ldots ,Z_{n}$. Let $Z_{0,n}=a:=\inf\left\{z:F\left(z\right)>0\right\}$ and $Z_{n+1,n}=b:=\sup\left\{z:F\left(z\right)<1\right\}$ denote the end-points of the common distribution of these observations, and assume that the density *f* is Riemann integrable and bounded away from 0 over each interval $\left[a^{\prime },b^{\prime }\right]\subset \left(a,b\right)$. For a specified k≥1, we establish the asymptotic normality of the sum of logarithms of the *k*-spacings $Z_{i+k,n}-Z_{i-1,n}$ for i=1,…,n−k+2. Our results complete previous investigations in the literature conducted by Blumenthal, Cressie, Shao and Hahn, and the references therein.

## 1. Introduction and Results

Let $X_{1},X_{2},\ldots$ be a sequence of independent replicæ of a non-degenerate random variable *X*, with distribution function F(x):=P(X≤x), defined for $x\in \bar{R}:=R\cup \left\{-\infty \right\}\cup \left\{-\infty \right\}$. We denote by a:=inf{x:F(x)>0}, and b:=sup{x:F(x)<1}, the distribution end-points, and we assume that a version of the the density $f\left(x\right)=\frac{d}{dx}F\left(x\right)$ of *X* exists for x∈R and is Riemann integrable and bounded away from 0 over each interval $\left[a^{\prime },b^{\prime }\right]\subset \left(a,b\right)$. For each n≥0, we set $X_{0,n}:=a$ and $X_{n+1,n}:=b$, and for each n≥1, we denote the order statistics of $X_{1},\ldots ,X_{n}$ by $X_{1,n},\ldots ,X_{n,n}$, which fulfill almost surely the strict inequalities

(1) $$-\infty \leq a=X_{0,n}<X_{1,n}<\ldots <X_{n,n}<b=X_{n+1,n}\leq \infty .$$

Given a specified integer k≥1, we are concerned with the limiting behavior as n→∞ of the sums of the logarithms of the *k*-spacings $\left\{D_{i,n}^{\left(k\right)}:1\leq i\leq n-k+2\right\}$, defined for n≥k−1 by

(2) $$D_{i,n}^{\left(k\right)}:=X_{i+k-1,n}-X_{i-1,n}\;\mathrm{for}\;i=1,\ldots ,n-k+2.$$

Since the first and last among the *k*-spacings in (2), namely, $D_{1,n}^{\left(k\right)}$ and $D_{n-k+2,n}^{\left(k\right)}$, are possibly infinite, we set

(3) $$\begin{array}{ccc} p_{0} & := & \left\{\begin{array}{cc} 1 & \mathrm{when}\;a>-\infty ,\\ 2 & \mathrm{when}\;a=-\infty , \end{array}\right. \end{array}$$

(4) $$\begin{array}{ccc} q_{0} & := & \left\{\begin{array}{cc} 1 & \mathrm{when}\;b<\infty ,\\ 2 & \mathrm{when}\;b=\infty , \end{array}\right. \end{array}$$

and consider only the finite *k*-spacings $\left\{D_{i,n}^{\left(k\right)}:p_{0}\leq i\leq n-k+3-q_{0}\right\}$.

Our goal is to investigate the limiting behavior, as n→∞, of the statistic

(5) $$\begin{array}{c} T_{n}\left(k;p,q\right):=\sum_{i=p}^{n-k+3-q}\left\{-\log\left(\frac{n}{k}\;D_{i,n}^{\left(k\right)}\right)\right\}, \end{array}$$

defined for each set of integers k≥1, $p\geq p_{0}$, $q\geq q_{0}$, and n≥k+p+q−3. In the present paper, we establish the asymptotic normality of $T_{n}\left(k;p,q\right)$ when *k*, *p*, and *q* are fixed, and n→∞. The motivation of these statistics is to provide tests of the goodness of fit of the null hypothesis (H.0) that *X* is uniformly distributed on (0,1) with $f\left(x\right)=1\;I_{\left(0,1\right)}\left(x\right)$, against the alternative. Darling [1] introduced the statistic $T_{n}:=T_{n}\left(1;2,2\right)$, and, later, Blumenthal [2] showed that, under the assumption that *f* is continuous on (a,b)⊂(−∞,∞) and bounded away from 0 on this interval, we have, as n→∞

(6) $$\begin{array}{c} n^{1/2}\left\{T_{n}-n\gamma -nE\left(\log f(X)\right)\right\}\overset{d}{\to }N\left(0,\zeta \left(2\right)+1+\mathrm{Var}\left(\log f(X)\right)\right). \end{array}$$

Here, and in the sequel, we write “$\overset{d}{\to }$” to denote weak convergence and “$\overset{d}{=}$” to denote equality in distribution. We let $N(\mu ,\sigma^{2})$ stand for the Gaussian distribution with mean μ and variance $\sigma^{2}$. Throughout, we use the convention that $\sum_{\emptyset }\left(\cdot \right):=0$ and $\prod_{\emptyset }\left(\cdot \right):=1$. We denote by γ=0.577215… *Euler’s constant* (see, e.g., (14) below) and set $\zeta \left(2\right)=\frac{\pi^{2}}{6}$ for the value taken by the *Riemann zeta function* ζ(z) for z=2 (we refer to Remark 2 in the sequel for some basic facts concerning these mathematical objects).

Under the null hypothesis (H.0) that *X* is uniformly distributed on (0,1), which implies that f(X)=1 a.s., (6) reduces to

(7) $$\begin{array}{c} n^{1/2}\left\{T_{n}-n\gamma \right\}\overset{d}{\to }N\left(0,\zeta (2)+1\right). \end{array}$$

For 0<α<1, denote by $\nu_{\alpha }$ the upper α quantile of the N(0,1) distribution. It follows from (6) that the test rejecting (H.0) when $n^{1/2}\left\{T_{n}-n\gamma \right\}\geq \nu_{\alpha }\sqrt{\zeta (2)+1}$ is asymptotically consistent with size α when n→∞. This result may be refined by using the exact distribution of $n^{1/2}\left\{T_{n}-n\gamma \right\}$, which was obtained in tractable form by Deheuvels and Derzko [3]. The corresponding results allow for the practical use of the so-called Darling–Blumenthal test of uniformity.

Some practical problems arise for the use of the above-described test in the presence of *ties* (see, e.g., pp. 118–124 in Hájek and Šidák [4]) when some of the observed spacings in the sequence $\left\{D_{i;n}^{\left(1\right)}:2\leq i\leq n\right\}$ are null, in which case $T_{n}$ is not properly defined. In practice, the use of the *k*-spacings $\left\{D_{i;n}^{\left(k\right)}:2\leq i\leq n-k+1\right\}$ for the choice of k≥1 that is sufficiently large allows us to overcome this difficulty. This motivates the study of the limiting behavior $T_{n}\left(k;p,q\right)$ for a specified choice of the integer k≥1.

We work under the assumptions listed in (F.1–3) below:

(*F*.1) $\mathrm{Var}\left(\log f(X)\right)<\infty$;
(*F*.2) Either a>−∞ and *f* is Riemann integrable and bounded away from 0 in a right neighborhood of *a*, or
  a≥−∞ and *f* is monotone in a right neighborhood of *a*;
(*F*.3) Either b<∞ and *f* is Riemann integrable and bounded away from 0 in a left neighborhood of *b*, or
  b≤∞ and *f* is monotone in a left neighborhood of *b*.

Our main result is stated in Theorem 1 below. We set $\psi \left(z\right):=\psi^{\left(1\right)}\left(z\right)=\frac{d}{dz}\log\Gamma \left(z\right)$ for the *digamma function* (see Remark 2 in the sequel for details on Euler’s constant γ, Riemann’s ζ(·) function, the *Gamma function* Γ(·), and the *polygamma functions* $\psi^{\left(m\right)}\left(\cdot \right)$ for m=0,1,…).

**Theorem** **1.** *Under (F.1–*2*–3), for each specified set of integers k≥1, $p\geq p_{0}$, and $q\geq p_{0}$, we have, as n→∞,*

(8) $$\begin{array}{c} n^{-1/2}\left[T_{n}\left(k;p,q\right)-n\left\{-\psi \left(k\right)+\log k+E\left(\log f(X)\right)\right\}\right]\\ \;\overset{d}{\to }\;N\left(0,\psi^{\prime }\left(k\right)+1+\mathrm{Var}\left(\log f(X)\right)\right). \end{array}$$

**Remark** **1.** *Under the assumptions above, for any specified set of integers k≥1, $p^{\prime },p^{\prime \prime }\geq p_{0}$, and $q^{\prime },q^{\prime \prime }\geq p_{0}$, we have*

(9) $$|T_{n}\left(k;p^{\prime },q^{\prime }\right)-T_{n}\left(k;p^{\prime \prime },q^{\prime \prime }\right)|=O_{P}\left(1\right)\;\mathrm{as}\;n\to \infty .$$

*Therefore, when the conclusion of Theorem 1 holds for some specified pairs of integers (p,q) with $p\geq p_{0}$ and $q\geq q_{0}$, it holds for all other pairs of integers p,q, fulfilling this condition. Because of this, we set $T_{n}\left(k\right):=T_{n}\left(k;p_{0},q_{0}\right)$ and give a proof of the theorem for $p=p_{0}$ and $q=q_{0}$.*

The proof of Theorem 1 is given in the next section, together with additional results of interest. We mention at this point some historical details about the sums of the logarithms of spacings and related topics. The study of spacings has received considerable attention in the literature, ever since the pioneering work of Darling [1] (see, e.g., Pyke [5,6], Deheuvels [7], and the references therein). To the best of our knowledge, the best result coming close to Theorem 1 was obtained by Cressie [8,9] for p=q=2 and k≥1. Cressie established a variant of (8) under the assumption that the density *f* of *X* is a bounded step function (see Theorem 5.1, p. 352 in [8]). For p=q=2 and k=1, a version of Theorem 1 was given by Blumenthal [2] under rather strenuous conditions on *f*, assumed to be, at least, twice differentiable. Shao and Hahn [10] largely improved Blumenthal’s theorem by showing that (8) holds for p=q=1, k=1, and when −∞<a<b<∞ under the assumption that *f* is Riemann integrable and bounded away from 0 on (a,b). Recently, Deheuvels and Derzko [11] (see also [3]) relaxed the assumptions of Blumenthal and Shao and Hahn by giving a version of Theorem 1 for k=1, allowing *a* and *b* to be possibly infinite. The present paper improves these results by covering the case of an arbitrary k≥1 under less strenuous conditions on *f*. We refer to Shao and Hahn [10], del Pino [12,13], Czekała [14], and the references therein for discussions and further references on the statistical applications of this theorem.

**Remark** **2.** ($1^{\circ }$) *The following relations and definitions hold, relating Euler’s constant γ=0.577215… to the Riemann zeta function ζ(·) in (6) (see, e.g., Spanier and Oldham [15] and Gradshteyn and Ryzhik [16], p. xxix). We have, for each r>1 and m=1,2,…,*

(10) $$\begin{array}{ccc} & & \zeta \left(r\right):=\frac{1}{\Gamma (r)}\int_{0}^{\infty }\frac{t^{r-1}dt}{e^{t}-1}=\sum_{j=1}^{\infty }\frac{1}{j^{r}}\;\mathrm{for}\;r>1, \end{array}$$

(11) $$\begin{array}{ccc} & & \zeta \left(2m\right):=\frac{2^{2m}\pi^{2m}B_{2m}}{(2m)!}, \end{array}$$

*where B_n_ is the n-th Bernoulli number. In particular, for r = 2 and m = 1,*

(12) $$\begin{array}{c} \zeta \left(2\right)=\frac{\pi^{2}}{6}=\frac{1}{\Gamma (2)}\int_{0}^{\infty }\frac{tdt}{e^{t}-1}=\sum_{j=1}^{\infty }\frac{1}{j^{2}}. \end{array}$$

*The Bernoulli numbers {Bn : n ≥ 0} are defined as the constants in the expansion*

(13) $$\begin{array}{c} \frac{t}{e^{t}-1}=\sum_{n=0}^{\infty }B_{n}\frac{t^{n}}{n!}\;\mathrm{which}\;\mathrm{converges}\;\mathrm{for}\;\left|t\right|<2\pi . \end{array}$$

*The Euler constant γ may be defined by either one of the relations*

(14) $$\begin{array}{c} \gamma :=\int_{0}^{\infty }\left(-\log t\right)e^{-t}dt=\lim_{r↓1}\left\{\zeta \left(r\right)-\frac{1}{r-1}\right\}\\ \;=\lim_{n\to \infty }\left\{\sum_{j=1}^{n-1}\frac{1}{j}-\log n\right\}. \end{array}$$

($2^{\circ }$) *The Euler Gamma function Γ(·), digamma function ψ(·), and polygamma function $\psi^{\left(m\right)}\left(\cdot \right)$ are respectively defined via the relations (see, e.g., §§6.3–6.4 in Abramowitz and Stegun [17]) for z>0 and m≥1,*

(15) $$\begin{array}{ccc} & & \Gamma \left(z\right):=\int_{0}^{\infty }t^{z-1}e^{-t}dt, \end{array}$$

(16) $$\psi \left(z\right):=\frac{d}{dz}\log\Gamma \left(z\right)=-\gamma +\int_{0}^{\infty }\frac{e^{-t}-e^{-zt}}{1-e^{-t}}\;dt,$$

*which fulfills*

$$\lim_{z\to \infty }\left\{\psi (z)-\log z\right\}=0,$$

(17) $$\begin{array}{ccc} & & \psi^{\left(m\right)}\left(z\right):=\frac{d^{m+1}}{dz^{m+1}}\log\Gamma \left(z\right)={\left(-1\right)}^{m+1}\int_{0}^{\infty }\frac{t^{m}e^{-zt}}{1-e^{-t}}\;dt. \end{array}$$

*In particular, when z=n≥1 is an integer, we obtain (see, e.g., Formulas 6.3.2 and 6.4.2 in [17])*

$$\begin{array}{ccc} & & \Gamma \left(n\right)=\left(n-1\right)!=\prod_{j=1}^{n-1}j, \end{array}$$

(18) $$\begin{array}{c} \psi \left(n\right):=-\gamma +\sum_{j=1}^{n-1}\frac{1}{j}\;,\;\psi \left(1\right)=-\gamma , \end{array}$$

*which fulfills*

(19) $$\begin{array}{c} \lim_{n\to \infty }\left\{\psi (n)-\log n\right\}=0,\\ \psi^{\left(m\right)}\left(n\right):={\left(-1\right)}^{m+1}m!\left\{\zeta \left(m+1\right)-\sum_{j=1}^{n-1}\frac{1}{j^{m+1}}\right\}, \end{array}$$

(20) $$\begin{array}{c} \psi^{\prime }\left(n\right):=\zeta \left(2\right)-\sum_{j=1}^{n-1}\frac{1}{j^{2}}=\sum_{j=n}^{\infty }\frac{1}{j^{2}}\\ =\int_{0}^{\infty }\frac{te^{-nt}}{1-e^{-t}}\;dt=\int_{0}^{1}\frac{u^{n-1}\left\{-\log u\right\}}{1-u}\;du. \end{array}$$

($3^{\circ }$) *Routine computations show that, as n→∞,*

$$\left\{\psi (n+1)-\log(n+1)\right\}-\left\{\psi (n)-\log n\right\}=\frac{1+o(1)}{2n^{2}},$$

*whence, as n→∞,*

(21) $$n\left\{\psi (n)-\log n\right\}\;\to \;-\frac{1}{2}\;.$$

($4^{\circ }$) *In view of (12) and (20), we readily obtain that, for k=1,2,…,*

$$\begin{array}{ccc} \psi^{\prime }\left(k\right) & = & \zeta \left(2\right)-\sum_{j=1}^{k-1}\frac{1}{j^{2}}=\sum_{j=k}^{\infty }\frac{1}{j^{2}}=\sum_{j=k}^{\infty }\frac{1}{j(j+1)}+\sum_{j=k}^{\infty }\left\{\frac{1}{j^{2}}-\frac{1}{j(j+1)}\right\}\\ & = & \frac{1}{k}+\sum_{j=k}^{\infty }\left\{\frac{1}{j^{2}}-\frac{1}{j(j+1)}\right\}=\frac{1}{k}+\sum_{j=k}^{\infty }\left\{\frac{1}{j^{2}\left(j+1\right)}\right\} \end{array}$$

$$\begin{array}{ccc} & = & \frac{1}{k}+\sum_{j=k}^{\infty }\left\{\frac{1}{j^{2}\left(j+1\right)}\right\}+\frac{1}{2k^{2}}-\frac{1}{2}\sum_{j=k}^{\infty }\left\{\frac{1}{j^{2}}-\frac{1}{{\left(j+1\right)}^{2}}\right\}\\ & = & \frac{1}{k}+\frac{1}{2k^{2}}+\sum_{j=k}^{\infty }\left\{\frac{1}{j^{2}\left(j+1\right)}-\frac{j+\frac{1}{2}}{j^{2}{\left(j+1\right)}^{2}}\right\}=\frac{1}{k}+\frac{1}{2k^{2}}+\frac{1}{2}\sum_{j=k}^{\infty }\left\{\frac{1}{j^{2}{\left(j+1\right)}^{2}}\right\}\\ & = & \frac{1}{k}+\frac{1}{2k^{2}}+\frac{1}{6k^{3}}-\frac{1}{6}\sum_{j=k}^{\infty }\left\{\left\{\frac{1}{j^{3}}-\frac{1}{{\left(j+1\right)}^{3}}\right\}-\left\{\frac{3j^{2}+3j}{j^{3}{\left(j+1\right)}^{3}}\right\}\right\}\\ & = & \frac{1}{k}+\frac{1}{2k^{2}}+\frac{1}{6k^{3}}-\frac{1}{6}\sum_{j=k}^{\infty }\frac{1}{j^{3}{\left(j+1\right)}^{3}}\;. \end{array}$$

*This, in turn, implies that, as k→∞,*

(22) $$\begin{array}{ccc} \left(2k^{2}-2k+1\right)\psi^{\prime }\left(n\right)-2k+1 & = & \left(2k^{2}-2k+1\right)\left\{\zeta \left(2\right)-\sum_{j=1}^{k-1}\frac{1}{j^{2}}\right\}-2k+1\\ & = & \frac{1}{3k}+\frac{1}{6k^{2}}+\frac{1+o(1)}{6k^{3}}\to 0. \end{array}$$

*so that the limiting variance of $n^{-1/2}T_{n}\left(k;p,q\right)$ in (8) equals*

$$\begin{array}{c} \mathrm{Var}\left(\log f(X)\right)+\frac{1+o(1)}{3k}\to \;\mathrm{Var}\left(\log f(X)\right), \end{array}$$

*as k→∞.*

($5^{\circ }$) *Likewise, we infer from (14) that, as k→∞,*

$$\begin{array}{c} \psi \left(k\right)=\gamma -\sum_{j=1}^{k-1}\frac{1}{j}+\log k=\sum_{j=k}^{\infty }\left\{\log\left(1+\frac{1}{j}\right)-\frac{1}{j}\right\}=-\frac{1+o(1)}{k}, \end{array}$$

*so that the limiting centering factor of $n^{-1}T_{n}\left(k;p,q\right)$ in (8) equals*

$$\begin{array}{c} E\left(\log f(X)\right)-\frac{1+o(1)}{k}\to E\left(\log f(X)\right), \end{array}$$

*as k→∞. By all this, for large specified values of k, $n^{-1}T_{n}\left(k;p,q\right)$ follows approximatively, as n→∞, a normal distribution, with expectation $E\left(\log f(X)\right)$ and variance $n^{-1/2}\mathrm{Var}\left(\log f(X)\right)$. This gives a heuristical motivation for the use of the statistic $n^{-1}T_{n}\left(k;p,q\right)$ (taken with specified large values of k) to estimate the factor $E\left(\log f(X)\right)$.*

## 2. Proofs

### 2.1. Properties of the Gauss Hypergeometric Function

In our proofs, we make use of a series of identities related to hypergeometric functions, which are of independent interest. For any x∈R and n∈IN, define the *Pochhammer function* by

(23) $${\left(x\right)}_{n}:=x\left(x+1\right)\ldots \left(x+n-1\right)\;\mathrm{for}\;n=1,2,\ldots ,\;\mathrm{and}\;{\left(x\right)}_{0}:=1.$$

We note that, whenever x>0 and n∈IN:={0,1,…},

(24) $${\left(x\right)}_{n}=\frac{\Gamma (x+n)}{\Gamma (x)}.$$

In particular, we have ${\left(1\right)}_{n}=n!$ for each n∈IN. We refer to 18:3:1; 18:3:2, and 18:10:1 in Spanier and Oldham [15] for additional properties of the Pochhammer function. Recalling (16), (23) and (24), we obtain readily that, for −x∉{0,1,…,n−1}, ${\left(x\right)}_{n}\neq 0$ and

(25) $$\begin{array}{ccc} & & \frac{d}{dx}{\left(x\right)}_{n}={\left(x\right)}_{n}\sum_{j=0}^{n-1}\frac{1}{x+j}={\left(x\right)}_{n}\left\{\psi (x+n)-\psi (x)\right\}, \end{array}$$

(26) $$\begin{array}{ccc} & & \frac{d^{2}}{dx^{2}}{\left(x\right)}_{n}={\left(x\right)}_{n}\left\{\psi^{\prime }\left(x+n\right)-\psi^{\prime }\left(x\right)+{\left\{\psi (x+n)-\psi (x)\right\}}^{2}\right\}. \end{array}$$

The usual Gauss hypergeometric function is defined for c∉−IN and z∈C with |z|<1 by

(27) F(a,b;c;z):=F12(a,b;c;z):=∑m=0∞(a)m(b)m(c)mzmm!.

The function F(a,b;c;z) is defined for |z|=1 when Re(c−a−b)>0 (see, e.g., Ch.4 in Rainville). In particular (see, e.g., 60:7:2 in [15]), when this condition holds,

(28) $$F\left(a,b;c;1\right)=\frac{\Gamma (c)\Gamma (c-b-a)}{\Gamma (c-a)\Gamma (c-b)}.$$

In particular, we have

(29) $$F\left(1,b;c;1\right)=\sum_{m=1}^{\infty }\frac{{\left(b\right)}_{m}}{m{\left(c\right)}_{m}}=\frac{\Gamma (c)\Gamma (c-b-1)}{\Gamma (c-1)\Gamma (c-b)}=\frac{c-1}{c-b-1}.$$

The general hypergeometric function of order (p,q) is defined for integer p≤q+1 by

(30) Fqp(a1,…,ap;b1,…,bq;z):=∑m=0∞(a1)m…(ap)m(b1)m…(bq)mzmm!.

The following identity relates higher-order hypergeometric functions to lower-order ones. We have

Fq+1p+1(a1,…,ap,r;b1,…,bq,s;z)=Γ(r)Γ(s)Γ(s−r)∫01tr−1(1−t)s−r−1Fqp(a1,…,ap;b1,…,bq;tz)dt.

In particular,

F23(1,b,r;c,s;1)=∑m=1∞(b)m(r)mm(c)m(s)m=Γ(s)Γ(r)Γ(r−s)∫01tr−1(1−t)s−r−1F(1,b;c,t)dt=Γ(s)Γ(r)Γ(s−r)∫01tr−1(1−t)s−r−1∑m=1∞(b)mm(c)mtm.

For a>1, b>1, c>1, and 0≤x<1, we have (see, e.g., 60:10:3 in [15])

(31) $$\int_{0}^{x}F\left(a,b;c;t\right)dt=\frac{c-1}{\left(a-1)(b-1\right)}\left\{F(a-1,b-1;c-1;x)-1\right\}.$$

**Proposition** **1.** *We have, for c>b+1, c∉−IN,*

(32) $$\begin{array}{ccc} & & \sum_{m=0}^{\infty }\frac{{\left(b\right)}_{m}}{{\left(c\right)}_{m}}=\frac{c-1}{c-b-1}, \end{array}$$

(33) $$\begin{array}{ccc} & & \sum_{m=0}^{\infty }\frac{{\left(b\right)}_{m}}{{\left(c\right)}_{m}}\left\{\psi (c+m)-\psi (c)\right\}=\frac{b}{{\left(c-b-1\right)}^{2}}, \end{array}$$

*and*

(34) $$\begin{array}{c} \sum_{m=0}^{\infty }\frac{{\left(b\right)}_{m}}{{\left(c\right)}_{m}}\left\{\psi (b+m)-\psi (b)\right\}=\frac{c-1}{{\left(c-b-1\right)}^{2}}. \end{array}$$

**Proof.**  By combining (27) with (28), taken with a=1, so that ${\left(1\right)}_{m}=m!$ and z=1, we obtain

$$\begin{array}{c} \sum_{m=0}^{\infty }\frac{{\left(b\right)}_{m}}{{\left(c\right)}_{m}}=F\left(1,b;c;1\right)=\frac{\Gamma (c)\Gamma (c-b-1)}{\Gamma (c-1)\Gamma (c-b)}=\frac{c-1}{c-b-1}, \end{array}$$

which is (32). By combining (25) with (32), we obtain, in turn, that

$$\begin{array}{ccc} & & \frac{\partial }{\partial c}\left\{\sum_{m=0}^{\infty }\frac{{\left(b\right)}_{m}}{{\left(c\right)}_{m}}\right\}=\sum_{m=0}^{\infty }\frac{\partial }{\partial c}\left\{\frac{{\left(b\right)}_{m}}{{\left(c\right)}_{m}}\right\}=-\sum_{m=0}^{\infty }\frac{{\left(b\right)}_{m}}{{\left(c\right)}_{m}}\left\{\psi (c+m)-\psi (c)\right\}\\ & & =\frac{\partial }{\partial c}\left\{\frac{c-1}{c-b-1}\right\}=\frac{-b}{{\left(c-b-1\right)}^{2}}\;, \end{array}$$

which is (33). The proof of (34) follows along the same lines with the formal replacement of $\frac{\partial }{\partial c}$ by $\frac{\partial }{\partial b}$. Namely, we obtain

$$\begin{array}{ccc} & & \frac{\partial }{\partial b}\left\{\sum_{m=0}^{\infty }\frac{{\left(b\right)}_{m}}{{\left(c\right)}_{m}}\right\}=\sum_{m=0}^{\infty }\frac{\partial }{\partial b}\left\{\frac{{\left(b\right)}_{m}}{{\left(c\right)}_{m}}\right\}=\sum_{m=0}^{\infty }\frac{{\left(b\right)}_{m}}{{\left(c\right)}_{m}}\left\{\psi (b+m)-\psi (b)\right\}\\ & & =\frac{\partial }{\partial b}\left\{\frac{c-1}{c-b-1}\right\}=\frac{c-1}{{\left(c-b-1\right)}^{2}}\;, \end{array}$$

which is (34). □

**Proposition** **2.** *We have, for c>b>0,*

(35) $$\begin{array}{ccc} & & \sum_{m=1}^{\infty }\frac{{\left(b\right)}_{m}}{m{\left(c\right)}_{m}}=\psi \left(c\right)-\psi \left(c-b\right), \end{array}$$

(36) $$\begin{array}{ccc} & & \sum_{m=1}^{\infty }\frac{{\left(b\right)}_{m}}{m{\left(c\right)}_{m}}\left\{\psi (c+m)-\psi (c)\right\}=\psi^{\prime }\left(c\right)-\psi^{\prime }\left(c-b\right), \end{array}$$

*and*

(37) $$\begin{array}{c} \sum_{m=1}^{\infty }\frac{{\left(b\right)}_{m}}{m{\left(c\right)}_{m}}\left\{\psi (b+m)-\psi (b)\right\}=\psi^{\prime }\left(c-b\right). \end{array}$$

**Proof.**  By (27) and (28), we have, for 0≤x<1,

$$\begin{array}{ccc} & & \frac{c}{hb}\left\{F(h,b;c;x)-1\right\}=\frac{c}{hb}\sum_{m=1}^{\infty }\frac{{\left(h\right)}_{m}{\left(b\right)}_{m}}{{\left(c\right)}_{m}}\;\frac{x^{m}}{m!}\\ & & =\sum_{m=0}^{\infty }\frac{{\left(1+h\right)}_{m}{\left(1+b\right)}_{m}}{{\left(1+c\right)}_{m}}\;\frac{x^{m+1}}{(m+1)!}. \end{array}$$

whence by letting x↑1 and making use of (28),

$$\begin{array}{ccc} & & \frac{c}{hb}\left\{F(h,b;c;1)-1\right\}=\frac{c}{hb}\left\{\frac{\Gamma (c)\Gamma (c-h-b)}{\Gamma (c-h)\Gamma (c-b)}-1\right\}\\ & & =\sum_{m=0}^{\infty }\frac{{\left(1+h\right)}_{m}{\left(b+1\right)}_{m}}{{\left(c+1\right)}_{m}}\;\frac{1}{(m+1)!}\;. \end{array}$$

When h↓0, we have, for each m≥1,

$$\frac{{\left(1+h\right)}_{m}}{(m+1)!}↓\frac{1}{m+1}\;,$$

so that

$$\begin{array}{ccc} & & \lim_{h↓0}\sum_{m=0}^{\infty }\frac{{\left(1+h\right)}_{m}{\left(b+1\right)}_{m}}{\left(m+1\right)!{\left(c+1\right)}_{m}}=\sum_{m=0}^{\infty }\frac{{\left(b+1\right)}_{m}}{{\left(c+1\right)}_{m}}\;\frac{1}{m+1}\\ & & =\frac{c}{b}\lim_{h↓0}\frac{1}{h}\left\{\frac{\Gamma (c)\Gamma (c-h-b)}{\Gamma (c-h)\Gamma (c-b)}-1\right\}. \end{array}$$

Next, we make use of (16), which yields the expansions

$$\begin{array}{ccc} \frac{\Gamma (c-h)}{\Gamma (c)} & = & 1-h(1+o(1))\psi (c)\;\mathrm{as}\;h↓0, \end{array}$$

and

$$\begin{array}{ccc} \frac{\Gamma (c-h-b)}{\Gamma (c-b)} & = & 1-h(1+o(1))\psi (c-b)\;\mathrm{as}\;h↓0. \end{array}$$

It follows readily that

$$\begin{array}{c} \lim_{h↓0}\frac{1}{h}\left\{\frac{\Gamma (c)\Gamma (c-h-b)}{\Gamma (c-h)\Gamma (c-b)}-1\right\}=\psi \left(c\right)-\psi \left(c-b\right). \end{array}$$

By all this, we obtain

$$\begin{array}{c} \sum_{m=0}^{\infty }\frac{{\left(b\right)}_{m}}{m{\left(c\right)}_{m}}=\frac{b}{c}\sum_{m=0}^{\infty }\frac{{\left(b+1\right)}_{m}}{{\left(c+1\right)}_{m}}\;\frac{1}{m+1}=\psi \left(c\right)-\psi \left(c-b\right), \end{array}$$

which is (35). Given (35), we infer from (25) and (35) that

$$\begin{array}{ccc} & & \psi^{\prime }\left(c\right)-\psi^{\prime }\left(c-b\right)=\frac{\partial }{\partial c}\left\{\psi (c)-\psi (c-b)\right\}\\ & & =\lim_{h↓0}\frac{1}{h}\sum_{m=1}^{\infty }\left\{\frac{{\left(b\right)}_{m}}{m{\left(c+h\right)}_{m}}-\frac{{\left(b\right)}_{m}}{m{\left(c\right)}_{m}}\right\}\\ & & =-\sum_{m=1}^{\infty }\frac{{\left(b\right)}_{m}}{m}\left\{\lim_{h↓0}\frac{1}{h}\left[\frac{1}{{\left(c+h\right)}_{m}}-\frac{1}{{\left(c\right)}_{m}}\right]\right\}\\ & & =\sum_{m=1}^{\infty }\frac{{\left(b\right)}_{m}}{m{\left(c\right)}_{m}^{2}}\frac{d}{dc}{\left(c\right)}_{m}=\sum_{m=1}^{\infty }\frac{{\left(b\right)}_{m}}{m{\left(c\right)}_{m}}\left\{\psi (c+m)-\psi (c)\right\}, \end{array}$$

which is (36). Likewise, we infer from (25) and (35) that

$$\begin{array}{ccc} & & \psi^{\prime }\left(c-b\right)=\frac{\partial }{\partial b}\left\{\psi (c)-\psi (c-b)\right\}=\lim_{h↓0}\frac{1}{h}\sum_{m=1}^{\infty }\left\{\frac{{\left(b+h\right)}_{m}}{m{\left(c\right)}_{m}}-\frac{{\left(b\right)}_{m}}{m{\left(c\right)}_{m}}\right\}\\ & & =\sum_{m=1}^{\infty }\frac{1}{m{\left(c\right)}_{m}}\left\{\lim_{h↓0}\frac{1}{h}\left[{\left(b+h\right)}_{m}-{\left(b\right)}_{m}\right]\right\}=\sum_{m=1}^{\infty }\frac{1}{m{\left(c\right)}_{m}}\frac{d}{db}{\left(b\right)}_{m}\\ & & =\sum_{m=1}^{\infty }\frac{{\left(b\right)}_{m}}{m{\left(c\right)}_{m}}\left\{\psi (b+m)-\psi (b)\right\}, \end{array}$$

which yields (37). □

### 2.2. Preliminary Results and Moment Calculations

The special case where *X* follows a uniform distribution on (0,1) will play an instrumental role in our proofs. For a general *F*, keeping in mind that the existence of $f\left(x\right)=\frac{d}{dx}F\left(x\right)$ implies that *F* is continuous, we set $U_{1}=F\left(X_{1}\right),U_{2}=F\left(X_{2}\right),\ldots$, and we observe that these random variables are independent, each with a uniform distribution on (0,1). For each n≥1, we denote by

(38) $$U_{0,n}:=0<U_{1,n}=F\left(X_{1,n}\right)<\ldots <U_{n,n}=F\left(X_{n,n}\right)<U_{n+1,n}:=1,$$

the order statistics of $0,1,U_{1},\ldots ,U_{n}$, with the convention that $U_{0,n}=F\left(X_{0,n}\right)=F\left(a\right)=0$ and $U_{n+1,n}=F\left(X_{n+1,n}\right)=F\left(b\right)=1$ for n≥0. We note that the inequalities in (38) hold a.s. We therefore assume that, without the loss of generality, they are fulfilled on the probability space on which $\left\{X_{n}:n\geq 1\right\}$ is defined. The *uniform k-spacings* are then given for 1≤k≤n+1 by

(39) $$\Delta_{i,n}^{\left(k\right)}=U_{i+k-1,n}-U_{i-1,n}\;\mathrm{for}\;i=1,\ldots ,n-k+2.$$

For r≥0, denote by $S_{r}\overset{d}{=}\Gamma \left(r\right)$ a random variable following a *Gamma distribution* with mean *r*. Namely, $S_{0}:=0$, and, for r>0, $S_{r}$ has density on R, given by

(40) $$h_{r}\left(s\right):=\left\{\begin{array}{cc} \frac{s^{r-1}}{\Gamma (r)}\;e^{-s} & \mathrm{for}\;s>0,\\ 0 & \mathrm{for}\;s\leq 0. \end{array}\right.$$

where $\Gamma \left(r\right):=\int_{0}^{\infty }s^{r-1}e^{-s}ds$ for r>0. When r=1, $S_{1}\overset{d}{=}\Gamma \left(1\right)$ is *exponentially distributed* with a unit mean. In this special case, we use the alternative notation $S_{1}\overset{d}{=}E\left(1\right)$. In general, for λ>0, we denote by $Z\overset{d}{=}E\left(\lambda \right)$ an exponentially distributed r.v. *Z* with mean 1/λ, fulfilling $\lambda Z\overset{d}{=}E\left(1\right)$. For p>0 and q>0, we denote by $R_{p,q}\overset{d}{=}\beta \left(p,q\right)$ a random variable following a *Beta distribution* with parameters *p* and *q*, meaning that $R_{p,q}$ has density given by

(41) $$g_{p,q}\left(s\right):=\left\{\begin{array}{cc} \frac{s^{p-1}{\left(1-s\right)}^{q-1}}{\beta (p,q)} & \mathrm{for}\;0<s<1,\\ 0 & \mathrm{for}\;s\notin (0,1). \end{array}\right.$$

The functions β(·,·) and Γ(·) are related by *Euler’s formula*. For any p>0 and q>0, we have

(42) $$\beta \left(p,q\right):=\int_{0}^{1}u^{p-1}{\left(1-u\right)}^{q-1}du=\frac{\Gamma (p)\Gamma (q)}{\Gamma (p+q)}\;.$$

We extend this definition when either p=0 or q=0 by setting

(43) $$R_{0,q}=0\overset{d}{=:}\beta \left(0,q\right)\;\left(q>0\right)\;\mathrm{and}\;R_{p,0}=1\overset{d}{=:}\beta \left(p,0\right)\;\left(p>0\right).$$

We refer to Ch. 17, 19, and 25 in Johnson, Kotz, and Balakrishnan [18,19] for useful details concerning the Gamma, exponential, and Beta distributions. In particular, we have the following useful distributional identity (see, e.g., p.12 in David [20]). For any 0≤j≤j+i≤n+1,

(44) $$U_{i+j,n}-U_{j,n}\overset{d}{=}\beta \left(i,n-i+1\right).$$

In particular, we have the distributional identity, for any 0≤i≤n+1,

(45) $$U_{i,n}\overset{d}{=}1-U_{n-i+1,n}\overset{d}{=}\beta \left(i,n-i+1\right).$$

The following lemma plays an instrumental role in our proofs.

**Lemma** **1.** *For p>0, q>0, and r>0, let $S_{p}\overset{d}{=}\Gamma \left(p\right)$, $S_{q}^{\prime }\overset{d}{=}\Gamma \left(q\right)$, and $S_{r}^{\prime \prime }\overset{d}{=}\Gamma \left(r\right)$ be three independent*

*Gamma-distributed r.v.’s. Then, the r.v.’s*

(46) $$R_{p,q}:=\frac{S_{p}}{S_{p}+S_{q}^{\prime }}\overset{d}{=}\beta \left(p,q\right)\;\mathrm{and}\;T_{p,q}:=S_{p}+S_{q}^{\prime }\overset{d}{=}\Gamma \left(p+q\right),$$

*are independent and follow β(p,q) and Γ(p+q) distributions, respectively. Set further*

(47) $$\begin{array}{ccc} R_{p,q,r}^{\prime } & := & \frac{S_{p}+S_{q}^{\prime }}{S_{p}+S_{q}^{\prime }+S_{r}^{\prime \prime }}\overset{d}{=}\beta \left(p+q,r\right), \end{array}$$

(48) $$\begin{array}{ccc} R_{p,q,r}^{\prime \prime } & := & \frac{S_{p}+S_{r}^{\prime \prime }}{S_{p}+S_{q}^{\prime }+S_{r}^{\prime \prime }}\overset{d}{=}\beta \left(p+r,q\right), \end{array}$$

*and*

(49) $$\begin{array}{ccc} T_{p,q,r}^{\prime } & := & S_{p}+S_{q}^{\prime }+S_{r}^{\prime \prime }\overset{d}{=}\Gamma \left(p+q+r\right). \end{array}$$

*Then, the r.v. $T_{p,q,r}^{\prime }$ is independent of the random pair $\left(R_{p,q,r}^{\prime },R_{p,q,r}^{\prime \prime }\right)$.*

**Proof.**  Several variants of the above results have been given in the literature (see, e.g., §25.2, p. 212 in Johnson, Kotz and Balakrishnan [19]). As the proofs are simple, we give details, limiting ourselves to (46). By the change of the variables u=s/(s+t) and v=s+t⇔s=uv and t=(1−u)v, the joint density of $\left(R_{p,q},T_{p,q}\right)$ is given by

$$\begin{array}{ccc} & & g\left(u,v\right)=h_{p}\left(uv\right)h_{q}\left(\left(1-u\right)v\right)|\det\left[\begin{array}{cc} \frac{\partial s}{\partial u} & \frac{\partial s}{\partial v}\\ \frac{\partial t}{\partial u} & \frac{\partial t}{\partial v} \end{array}\right]|\;\;\\ & & =\left\{\frac{v^{p+q-1}e^{-v}}{\Gamma (p+q)}\right\}\left\{\frac{u^{p-1}{\left(1-u\right)}^{q-1}}{\beta (p,q)}\right\}\left\{\frac{\beta (p,q)}{\Gamma (p)\Gamma (q)}\right\}\frac{1}{v}|\det\left[\begin{array}{cc} v & u\\ -v & 1-u \end{array}\right]|\\ & & =h_{p+q}\left(u\right)g_{p,q}\left(v\right), \end{array}$$

which is sufficient for our needs. □

In view of (38), set $Y_{1}=-\log U_{1},Y_{2}=-\log U_{2},\ldots$, and observe that $Y_{1},Y_{2},\ldots$ constitutes a sequence of independent E(1), unit mean exponential random variables. For each n≥1, the order statistics of $Y_{1},\ldots ,Y_{n}$ fulfill the relations

(50) $$\begin{array}{ccc} 0<Y_{1,n}=-\log U_{n,n}<\ldots & < & Y_{i,n}=-\log U_{n-i+1,n}<\ldots \\ & < & Y_{n,n}=-\log U_{1,n}<\infty . \end{array}$$

Set, for convenience, $Y_{0,n}=-\log U_{n+1,n}=0$ for n≥1. We will need the following useful fact, closely related to Lemma 1 (refer to Sukhatme [21] and Malmquist [22], and see, e.g., pp. 20–21 in David [20]):

**Fact** **1.** *For each* n≥1*, the random variables*

(51) $$\begin{array}{c} \omega_{i,n}:=\left(n-i+1\right)\left\{Y_{i,n}-Y_{i-1,n}\right\}\overset{d}{=}E\left(1\right),\;i=1,\ldots ,n, \end{array}$$

*are independent, each following an exponential* E(1) *distribution.*

It will be convenient, later on, to make use of the relation following from (51),

(52) $$Y_{i,n}=-\log U_{n-i+1,n}=\sum_{j=1}^{i}\frac{\omega_{j,n}}{n-j+1}\;,\;i=1,\ldots ,n.$$

In Lemma 2 below, we evaluate the moments of the logarithms of Gamma-distributed random variables, which will play an instrumental role later on. As usual, we make use of the convention that $\sum_{\emptyset }\left(\cdot \right)=0$.

**Lemma** **2.** *Let k≥1 be an integer, and let $S_{k}\overset{d}{=}\Gamma \left(k\right)$ be a Gamma-distributed random variable with mean k. Then, for each k≥1,*

(53) $$\begin{array}{ccc} \gamma_{k} & := & E\left(-\log S_{k}\right)=-\psi \left(k\right)=\gamma -\sum_{j=1}^{k-1}\frac{1}{j}, \end{array}$$

(54) $$\begin{array}{ccc} r_{k} & := & E\left(-S_{k}\log S_{k}\right)=-k\psi \left(k\right)-1\\ & = & k\gamma_{k}-1=k\gamma -k\sum_{j=1}^{k-1}\frac{1}{j}-1, \end{array}$$

*and*

(55) $$\begin{array}{ccc} s_{k} & := & E\left({\left\{-\log S_{k}\right\}}^{2}\right)=\psi {\left(k\right)}^{2}+\psi^{\prime }\left(k\right)\\ & = & \zeta \left(2\right)-\sum_{j=1}^{k-1}\frac{1}{j^{2}}+\gamma_{k}^{2}=\sum_{j=k}^{\infty }\frac{1}{j^{2}}+{\left\{\gamma -\sum_{j=1}^{k-1}\frac{1}{j}\right\}}^{2}\\ & = & \frac{\pi^{2}}{6}-\sum_{j=1}^{k-1}\frac{1}{j^{2}}+{\left\{\gamma -\sum_{j=1}^{k-1}\frac{1}{j}\right\}}^{2}. \end{array}$$

**Proof.**  Recalling the definition (40) of $h_{r}\left(s\right)$ for r>0 and the definition (53) of $\gamma_{k}$, we obtain that, by integrating by parts, for k≥2,

(56) $$\begin{array}{ccc} \gamma_{k} & = & \int_{0}^{\infty }\left\{-\log s\right\}h_{k}\left(s\right)ds=\int_{0}^{\infty }\left\{-\log s\right\}\frac{s^{k-1}}{(k-1)!}\;d\left\{-e^{-s}\right\}\\ & = & -{\left[\left\{-\log s\right\}\frac{s^{k-1}}{(k-1)!}\;e^{-s}\right]}_{s=0}^{s=\infty }\\ & & +\int_{0}^{\infty }\left\{-\log s\right\}\frac{s^{k-2}}{(k-2)!}\;d\left\{-e^{-s}\right\}\\ & & -\int_{0}^{\infty }\frac{s^{k-2}}{(k-1)!}\;e^{-s}ds=\gamma_{k-1}-\frac{1}{k-1}. \end{array}$$

For k=1, these relations reduce to (see, e.g., Formula 4.331, p. 573, in Gradshteyn and Ryzhik [16])

(57) $$\begin{array}{ccc} \gamma_{1} & = & \int_{0}^{\infty }\left\{-\log s\right\}h_{1}\left(s\right)ds=\int_{0}^{\infty }\left\{-\log s\right\}\;e^{-s}ds=\gamma . \end{array}$$

Recalling the definition (18) of ψ(m) for m=1,2,…, and the definition (53) of $\gamma_{k}$, by a straightforward induction on *k*, we infer from the above relations that, for an arbitrary (integer) k≥1,

(58) $$\begin{array}{ccc} \gamma_{k} & = & \gamma_{k-1}-\frac{1}{k-1}=\ldots =\gamma_{1}-\sum_{j=1}^{k-1}\frac{1}{j}\;=-\psi \left(k\right), \end{array}$$

which is (53). Likewise, in view of (54) and (56), by integrating by parts, we see that, for k≥2,

$$\begin{array}{ccc} r_{k} & = & \int_{0}^{\infty }s\left\{-\log s\right\}h_{k}\left(s\right)ds=\int_{0}^{\infty }\left\{-\log s\right\}\frac{s^{k}}{(k-1)!}\;d\left\{-e^{-s}\right\}\\ & = & -{\left[\left\{-\log s\right\}\frac{s^{k}}{(k-1)!}\;e^{-s}\right]}_{s=0}^{s=\infty }\\ & & +\int_{0}^{\infty }\left\{-\log s\right\}\frac{ks^{k-1}}{(k-1)!}\;d\left\{-e^{-s}\right\}\\ & & -\int_{0}^{\infty }\frac{s^{k-1}}{(k-1)!}\;e^{-s}ds=k\gamma_{k}-1=k\gamma -k\sum_{j=1}^{k-1}\frac{1}{j}-1, \end{array}$$

which is (54). In the same spirit, to establish (56), we integrate by parts to obtain the recursion formula, for k≥2,

(59) $$\begin{array}{ccc} s_{k} & = & \int_{0}^{\infty }{\left\{-\log s\right\}}^{2}h_{k}\left(s\right)ds=\int_{0}^{\infty }{\left\{-\log s\right\}}^{2}\frac{s^{k-1}}{(k-1)!}\;d\left\{-e^{-s}\right\}\\ & = & -{\left[{\left\{-\log s\right\}}^{2}\frac{s^{k-1}}{(k-1)!}\;e^{-s}\right]}_{s=0}^{s=\infty }\\ & & +\int_{0}^{\infty }{\left\{-\log s\right\}}^{2}\frac{s^{k-2}}{(k-2)!}\;d\left\{-e^{-s}\right\}\\ & & -\frac{2}{k-1}\int_{0}^{\infty }\left\{-\log s\right\}\frac{s^{k-2}}{(k-2)!}\;d\left\{-e^{-s}\right\}\\ & = & s_{k-1}-\frac{2\gamma_{k-1}}{k-1}\;. \end{array}$$

By combining (58) with (60), we readily obtain that

(60) $$\begin{array}{ccc} s_{k}-\gamma_{k}^{2} & = & s_{k}-{\left\{\gamma_{k-1}-\frac{1}{k-1}\right\}}^{2}\\ & = & s_{k-1}-\frac{2\gamma_{k-1}}{k-1}-{\left\{\gamma_{k-1}-\frac{1}{k-1}\right\}}^{2}\\ & = & s_{k-1}-\gamma_{k-1}^{2}-{\left\{\frac{1}{k-1}\right\}}^{2}\\ & = & s_{k-2}-\gamma_{k-2}^{2}-{\left\{\frac{1}{k-2}\right\}}^{2}-{\left\{\frac{1}{k-1}\right\}}^{2}\\ & = & \ldots =s_{1}-\gamma_{1}^{2}-\sum_{j=1}^{k-1}\frac{1}{j^{2}}. \end{array}$$

For k=1, we combine (57) with the fact that (see, e.g., Formula 4.335, p. 574, in Gradshteyn and Ryzhik [16])

(61) $$s_{1}-\gamma_{1}^{2}=\int_{0}^{\infty }{\left\{-\log s\right\}}^{2}e^{-s}ds-\gamma^{2}=\frac{\pi^{2}}{6}=\zeta \left(2\right)=\sum_{j=1}^{\infty }\frac{1}{j^{2}}.$$

In view of (53), (60) and (61), the relation (56) is straightforward. □

**Lemma** **3.** *Let $S_{k}\overset{d}{=}\Gamma \left(k\right)$ denote a Gamma-distributed random variable with expectation k. Then, for each integer k≥1, we have*

(62) $$\begin{array}{ccc} \sigma_{k}^{2} & := & \mathrm{Var}\left(-\log S_{k}\right)=s_{k}-\gamma_{k}^{2}=\psi^{\prime }\left(k\right)\\ & = & \zeta \left(2\right)-\sum_{i=1}^{k-1}\frac{1}{i^{2}}=\frac{\pi^{2}}{6}-\sum_{i=1}^{k-1}\frac{1}{i^{2}}=\sum_{i=k}^{\infty }\frac{1}{i^{2}}. \end{array}$$

**Proof.**  Even though the relation (62) is a direct consequence of (53) and (56), below, we give an alternate proof of this statement based upon Lemma 1. The corresponding arguments will be instrumental for the proof of the forthcoming Proposition 3. We may write, making use of (46) in Lemma 3, for each integer m≥1 and ℓ≥1,

$$\begin{array}{c} -\log S_{m}=-\log\left(\frac{S_{m}}{S_{m}+S_{\ell }^{\prime }}\right)+\log\left(S_{m}+S_{\ell }^{\prime }\right)=:V_{1}-V_{2}, \end{array}$$

where

(63) $$V_{1}:=-\log\left(\frac{S_{m}}{S_{m}+S_{\ell }^{\prime }}\right)\overset{d}{=}-\log Z,$$

with $Z\overset{d}{=}\beta \left(m,\ell \right)$, and

(64) $$V_{2}:=-\log\left(S_{m}+S_{\ell }^{\prime }\right)\overset{d}{=}-\log Y,$$

with $Y\overset{d}{=}\Gamma \left(m+\ell \right)$, are independent r.v.’s. Following the arguments of Lemma 1, we note that, in the above relations, $S_{m}\overset{d}{=}\Gamma \left(m\right)$ and $S_{\ell }^{\prime }\overset{d}{=}\Gamma \left(\ell \right)$ are two independent Gamma-distributed random variables, with expectations equal to *m* and *ℓ*, respectively. In view of (50), by combining (44) with (45) and (46), we readily obtain that

(65) $$\begin{array}{ccc} V_{1} & = & -\log\left(\frac{S_{m}}{S_{m}+S_{\ell }^{\prime }}\right)\\ & \overset{d}{=} & -\log U_{m,m+\ell -1}=Y_{\ell ,m+\ell -1}=\sum_{j=1}^{\ell }\frac{\omega_{j,m+\ell -1}}{m+\ell -j}\\ & \overset{d}{=} & -\log\left(1-U_{\ell ,m+\ell -1}\right)=-\log\left(1-\exp(-Y_{m,m+\ell -1})\right)\\ & = & -\log\left(1-\exp\left\{-\sum_{j=1}^{m}\frac{\omega_{j,m+\ell -1}}{m+\ell -j}\right\}\right). \end{array}$$

Set for convenience $\rho_{p}:=\sigma_{p}^{2}=\mathrm{Var}\left(-\log S_{p}\right)$, with $S_{p}\overset{d}{=}\Gamma \left(p\right)$ for p≥1; we infer from (63) and (64) that

(66) $$\begin{array}{ccc} \rho_{m} & = & \mathrm{Var}\left(V_{2}\right)+\mathrm{Var}\left(V_{1}\right)=\rho_{m+\ell }+\mathrm{Var}\left(V_{1}\right)\\ & = & \rho_{m+\ell }+\sum_{j=1}^{\ell }\frac{1}{{\left(m+\ell -j\right)}^{2}}=\rho_{m+\ell }+\sum_{i=m}^{m+\ell -1}\frac{1}{i^{2}}. \end{array}$$

By combining (57) and (61) with (62), we see that $\rho_{1}=\zeta \left(2\right)$. Therefore, (62) follows readily from (66), taken with m=1 and m+ℓ=k. □

**Lemma** **4.** *Let $Z\overset{d}{=}\beta \left(m,\ell \right)$, where m≥1 and ℓ≥1 are integers. Then,*

(67) $$\begin{array}{ccc} E(-\log Z) & = & \sum_{i=m}^{m+\ell -1}\frac{1}{i}=\psi \left(m+\ell \right)-\psi \left(m\right), \end{array}$$

*and*

(68) $$\begin{array}{ccc} \mathrm{Var}(-\log Z) & = & \sum_{i=m}^{m+\ell -1}\frac{1}{i^{2}}=\psi^{\prime }\left(m\right)-\psi^{\prime }\left(m+\ell \right). \end{array}$$

**Proof.**  We may write, by (19) and (20),

$$\begin{array}{ccc} E(-\log Z) & = & E\left(V_{1}\right)=E\left(\sum_{j=1}^{\ell }\frac{\omega_{j,m+\ell -1}}{m+\ell -j}\right)=\sum_{j=1}^{\ell }\frac{1}{m+\ell -j}\\ & = & \psi (m+\ell )-\psi (m), \end{array}$$

and

$$\begin{array}{ccc} \mathrm{Var}(-\log Z) & = & \mathrm{Var}\left(V_{1}\right)=\mathrm{Var}\left(\sum_{j=1}^{\ell }\frac{\omega_{j,m+\ell -1}}{m+\ell -j}\right)=\sum_{j=1}^{\ell }\frac{1}{{\left(m+\ell -j\right)}^{2}}\\ & = & \psi^{\prime }\left(m\right)-\psi^{\prime }\left(m+\ell \right), \end{array}$$

as sought. □

**Lemma** **5.** Let $\left\{\omega_{m}:m\geq 1\right\}$ denote an i.i.d. sequence of exponentially distributed E(1) random variables. For any m≥1, set $S_{m}=\omega_{1}+\ldots \omega_{m}\overset{d}{=}\Gamma \left(m\right)$ and $U_{m,n}^{*}:=S_{m}/S_{n+1}\overset{d}{=}\beta \left(m,n-m+1\right)$. Then, for any 1≤k≤n+1, we have

(69) $$\begin{array}{ccc} & & E\left(\left\{-\log S_{k}\right\}\left\{-\log S_{n+1}\right\}\right)=\psi \left(k\right)\psi \left(n+1\right)+\psi^{\prime }\left(n+1\right), \end{array}$$

*and*

(70) $$\begin{array}{c} \mathrm{Cov}\left(\left\{-\log S_{k}\right\},\left\{-\log S_{n+1}\right\}\right)=\psi^{\prime }\left(n+1\right). \end{array}$$

**Proof.**  We have

$$\begin{array}{ccc} & & E\left(\left\{-\log S_{k}\right\}\left\{-\log S_{n+1}\right\}\right)\\ = & & E\left(\left\{-\log\left(\frac{S_{k}}{S_{n+1}}\right)+\left\{-\log S_{n+1}\right\}\right\}\left\{-\log S_{n+1}\right\}\right)\\ = & & E\left(\left\{\left\{-\log U_{k,n}^{*}\right\}+\left\{-\log S_{n+1}\right\}\right\}\left\{-\log S_{n+1}\right\}\right)\}). \end{array}$$

Since $U_{k,n}^{*}$ and $S_{n+1}$ are independent, it follows that

$$\begin{array}{ccc} & & E\left(\left\{-\log S_{k}\right\}\left\{-\log S_{n+1}\right\}\right)\\ = & & E\left\{-\log U_{k,n}^{*}\right\}E\left\{-\log S_{n+1}\right\}+E\left({\left\{-\log S_{n+1}\right\}}^{2}\right). \end{array}$$

Making use of (67) and (57), we see that $E\left\{-\log U_{k,n}^{*}\right\}=\psi \left(n+1\right)-\psi \left(k\right)$, $E\left\{-\log S_{k}\right\}=-\psi \left(k\right)$, $E\left\{-\log S_{n+1}\right\}=-\psi \left(n+1\right)$, and $E\left({\left\{-\log S_{n+1}\right\}}^{2}\right)=\psi {\left(n+1\right)}^{2}+\psi^{\prime }\left(n+1\right)$. By all this, we obtain

$$\begin{array}{ccc} & & E\left(\left\{-\log S_{k}\right\}\left\{-\log S_{n+1}\right\}\right)\\ & & =-\psi {\left(n+1\right)}^{2}+\psi \left(k\right)\psi \left(n+1\right)+\psi {\left(n+1\right)}^{2}+\psi^{\prime }\left(n+1\right), \end{array}$$

which is (69). Given (69), the proof of (70) follows from the relations $E\left\{-\log S_{k}\right\}=-\psi \left(k\right)$ and $E\left\{-\log S_{n+1}\right\}=-\psi \left(n+1\right)$. We note that, when k=n+1, (69) yields

$$\begin{array}{ccc} & & E\left({\left\{-\log S_{n+1}\right\}}^{2}\right)=\psi {\left(n+1\right)}^{2}+\psi^{\prime }\left(n+1\right), \end{array}$$

which is in agreement with (56). □

**Proposition** **3.** *Let 0≤ℓ≤k be an integer, and let $S_{k-\ell }\overset{d}{=}\Gamma \left(k-\ell \right)$, $S_{\ell }^{\prime }\overset{d}{=}\Gamma \left(\ell \right)$, and $S_{\ell }^{\prime \prime }\overset{d}{=}\Gamma \left(\ell \right)$ be independent Gamma-distributed random variables. Then, we have*

(71) $$\begin{array}{ccc} s_{k,\ell } & := & E\left(\left\{-\log\{S_{k-\ell }+S_{\ell }^{\prime }\}\right\}\left\{-\log\{S_{k-\ell }+S_{\ell }^{\prime \prime }\}\right\}\right)\\ & = & \psi {\left(k\right)}^{2}-\psi^{\prime }\left(k+\ell \right)-{\left\{\psi (k+\ell )-\psi (k)\right\}}^{2}\\ & & +\sum_{m=1}^{\infty }\frac{{\left(\ell \right)}_{m}}{m{\left(k+\ell \right)}_{m}}\left\{\psi (k+\ell +m)-\psi (k+m)\right\}. \end{array}$$

**Proof.**  Set for convenience j=k−ℓ. When ℓ=0, we have $S_{\ell }^{\prime }=S_{\ell }^{\prime \prime }=0$, and, therefore, by (7), $s_{k,0}=\psi {\left(k\right)}^{2}\psi^{\prime }\left(k\right)$, which is in agreement with (71). Likewise, when ℓ=k, $S_{k-\ell }=0$, and, hence, $s_{k,k}=\gamma_{k}^{2}=\psi {\left(k\right)}^{2}$, which is also in agreement with (71). In fact, when k=ℓ, (71) may be rewritten into

(72) $$\begin{array}{ccc} s_{k,k} & = & \psi {\left(k\right)}^{2}-\psi^{\prime }\left(2k\right)-{\left\{\psi (2k)-\psi (k)\right\}}^{2}\\ & & +\sum_{m=1}^{\infty }\frac{{\left(k\right)}_{m}}{m{\left(2k\right)}_{m}}\left\{\psi (2k+m)-\psi (k+m)\right\},\\ & = & \psi {\left(k\right)}^{2}-\psi^{\prime }\left(2k\right)-{\left\{\psi (2k)-\psi (k)\right\}}^{2}\\ & & +\sum_{m=1}^{\infty }\frac{{\left(k\right)}_{m}}{m{\left(2k\right)}_{m}}\left\{\psi (2k+m)-\psi (2k)\right\}\\ & & +\left\{\psi (2k)-\psi (k)\right\}\sum_{m=1}^{\infty }\frac{{\left(k\right)}_{m}}{m{\left(2k\right)}_{m}}\\ & & -\sum_{m=1}^{\infty }\frac{{\left(k\right)}_{m}}{m{\left(2k\right)}_{m}}\left\{\psi (k+m)-\psi (2k)\right\}\\ & = & \psi {\left(k\right)}^{2}-\psi^{\prime }\left(2k\right)-{\left\{\psi (2k)-\psi (k)\right\}}^{2}\\ & & +\psi^{\prime }\left(2k\right)-\psi^{\prime }\left(k\right)+{\left\{\psi (2k)-\psi (k)\right\}}^{2}+\psi^{\prime }\left(k\right)=\psi {\left(k\right)}^{2}, \end{array}$$

where we have made use of (35), (36) and (37), taken with b=k and c=2k. Given that the values of $s_{k,0}=0$ and $s_{k,k}=\psi {\left(k\right)}^{2}$ are in agreement with (71), we may limit ourselves to establish this relation when *ℓ* and *j* fulfill 1≤ℓ,j≤k. In the remainder of our proof, we therefore assume that this condition is fulfilled.

We make use of the notation and conclusions of Lemma 1 to write that

(73) $$\begin{array}{ccc} \Sigma & := & E\left(\left\{-\log\{S_{j}+S_{\ell }^{\prime }\}\right\}\left\{-\log\{S_{j}+S_{\ell }^{\prime \prime }\}\right\}\right)\\ & = & E\left(\left\{-\log\{R_{j,\ell ,\ell }^{\prime }T_{j,\ell ,\ell }^{\prime }\}\right\}\left\{-\log\{R_{j,\ell ,\ell }^{\prime \prime }T_{j,\ell ,\ell }^{\prime }\}\right\}\right)\\ & = & E\left({\left\{-\log T_{j,\ell ,\ell }^{\prime }\right\}}^{2}\right)+E\left(-\log T_{j,\ell ,\ell }^{\prime }\right)\{E\left(-\log R_{j,\ell ,\ell }^{\prime }\right)\\ & + & E\left(-\log R_{j,\ell ,\ell }^{\prime \prime }\right)\}+E\left(\left\{-\log R_{j,\ell ,\ell }^{\prime }\right\}\left\{-\log R_{j,\ell ,\ell }^{\prime \prime }\right\}\right), \end{array}$$

where the random pair

(74) $$\begin{array}{ccc} R_{j,\ell ,\ell }^{\prime } & := & \frac{S_{j}+S_{\ell }^{\prime }}{S_{j}+S_{\ell }^{\prime }+S_{\ell }^{\prime \prime }}\overset{d}{=}\beta \left(j+\ell ,\ell \right), \end{array}$$

(75) $$\begin{array}{ccc} R_{j,\ell ,\ell }^{\prime \prime } & := & \frac{S_{j}+S_{\ell }^{\prime \prime }}{S_{j}+S_{\ell }^{\prime }+S_{\ell }^{\prime \prime }}\overset{d}{=}\beta \left(j+\ell ,\ell \right), \end{array}$$

is independent of

(76) $$\begin{array}{ccc} T_{j,\ell ,\ell }^{\prime } & := & S_{j}+S_{\ell }^{\prime }+S_{\ell }^{\prime \prime }\overset{d}{=}\Gamma \left(j+2\ell \right). \end{array}$$

Now, since $T_{j,\ell ,\ell }^{\prime }\overset{d}{=}S_{j+2\ell }\overset{d}{=}\Gamma \left(j+2\ell \right)\overset{d}{=}\Gamma \left(k+\ell \right)$, we infer from (53) and (56) that

(77) $$\begin{array}{ccc} E(-\log T_{j,\ell ,\ell }^{\prime }) & = & \psi (k+\ell ), \end{array}$$

and

(78) $$\begin{array}{ccc} E({\left\{-\log T_{j,\ell ,\ell }^{\prime }\right\}}^{2}) & = & \psi {\left(k+\ell \right)}^{2}-\psi^{\prime }\left(k+\ell \right). \end{array}$$

Next, set

$$\begin{array}{ccc} W_{1} & := & -\log R_{j,\ell ,\ell }^{\prime }=-\log\left(\frac{S_{j}+S_{\ell }^{\prime }}{S_{j}+S_{\ell }^{\prime }+S_{\ell }^{\prime \prime }}\right), \end{array}$$

and

$$\begin{array}{ccc} W_{2} & := & -\log R_{j,\ell ,\ell }^{\prime \prime }=-\log\left(\frac{S_{j}+S_{\ell }^{\prime \prime }}{S_{j}+S_{\ell }^{\prime }+S_{\ell }^{\prime \prime }}\right). \end{array}$$

We infer from (74) and (75), in combination with (67) and (68), taken with the formal change of (m,ℓ) into (j+ℓ,ℓ), that

(79) $$\begin{array}{ccc} E\left(W_{1}\right)=E\left(W_{2}\right) & = & E\left(-\log R_{j,\ell ,\ell }^{\prime }\right)=E\left(-\log R_{j,\ell ,\ell }^{\prime \prime }\right)\\ & = & \psi (j+2\ell )-\psi (j+\ell )=\psi (k+\ell )-\psi (k). \end{array}$$

By all this, we infer from (74), (77) and (78) that

(80) $$\begin{array}{ccc} \Sigma & = & \psi {\left(k+\ell \right)}^{2}-\psi^{\prime }\left(k+\ell \right)-2\psi \left(k+\ell \right)\left\{\psi (k+\ell )-\psi (k)\right\}\\ & & +E(W_{1}W_{2})\\ & = & \psi {\left(k\right)}^{2}-{\left\{\psi \left(k+\ell \right)-\psi \left(k\right)\right\}}^{2}-\psi^{\prime }\left(k+\ell \right)+E\left(W_{1}W_{2}\right). \end{array}$$

Next, we observe that the joint distribution of $\left(W_{1},W_{2}\right)$ coincides with that of $\left(W_{1}^{\left(*\right)},W_{2}^{\left(*\right)}\right)$, where

$$\begin{array}{ccc} W_{1}^{*} & = & -\log\left(U_{j+\ell ,j+2\ell -1}\right)\;,\\ W_{2}^{*} & = & -\log\left(1-U_{\ell ,j+2\ell -1}\right). \end{array}$$

We then observe that

$$\begin{array}{c} U:=\frac{U_{\ell ,j+2\ell -1}}{U_{j+\ell ,j+2\ell -1}}\overset{d}{=}U_{\ell ,j+\ell -1}\overset{d}{=}\beta \left(\ell ,j\right), \end{array}$$

is independent of $V:=U_{j+\ell ,j+2\ell -1}\overset{d}{=}\beta \left(j+\ell ,\ell \right)$. Given this fact, we make use of the Taylor expansion of

$$-\log\left(1-uv\right)=\sum_{m=1}^{\infty }\frac{u^{m}v^{m}}{m}\;\mathrm{for}\;\left|uv\right|<1,$$

to obtain that

(81) $$\begin{array}{ccc} & & E\left(W_{1}W_{2}\right)=E\left(W_{1}^{*}W_{2}^{*}\right)=E\left(\left\{-\log V\right\}\left\{-\log(1-UV)\right\}\right)\\ & = & \int_{0}^{1}\left\{\int_{0}^{1}\left\{-\log v\right\}\left\{-\log(1-uv)\right\}\frac{u^{\ell -1}{\left(1-u\right)}^{j-1}}{\beta (\ell ,j)}\;du\right\}\\ & & \times \frac{v^{j+\ell -1}{\left(1-v\right)}^{\ell -1}}{\beta (j+\ell ,\ell )}\;dv\\ & = & \sum_{m=1}^{\infty }\frac{1}{m}\left\{\int_{0}^{1}\frac{u^{\ell +m-1}{\left(1-u\right)}^{j-1}}{\beta (\ell ,j)}\;du\right\}\\ & & \times \int_{0}^{1}\left\{-\log v\right\}\left\{\frac{v^{j+\ell +m-1}{\left(1-v\right)}^{\ell -1}}{\beta (j+\ell ,\ell )}\right\}dv. \end{array}$$

Recall Euler’s formula β(r,s)=Γ(r)Γ(s)/Γ(r+s) and the definition of the Pochhammer symbol ${\left(a\right)}_{m}=\Gamma \left(a+m\right)/\Gamma \left(a\right)$ when a≠0 and a≠−m, and, in general,

$${\left(a\right)}_{m}=a\left(a+1\right)\ldots \left(a+m-1\right)\;\mathrm{for}\;m=1,2\ldots ,\;{\left(a\right)}_{0}=1.$$

Recalling that j+ℓ=k, we infer from (81) that

(82) $$\begin{array}{ccc} E(W_{1}W_{2}) & = & \sum_{m=1}^{\infty }\frac{\beta (\ell +m,j)\beta (j+\ell +m,\ell )}{m\beta (\ell ,j)\beta (j+\ell ,\ell )}\left\{\psi (k+\ell +m)-\psi (k+m)\right\}\\ & = & \sum_{m=1}^{\infty }\frac{\Gamma (\ell +m)\Gamma (k+\ell )}{m\Gamma (\ell )\Gamma (k+\ell +m)}\left\{\psi (k+\ell +m)-\psi (k+m)\right\}\\ & = & \sum_{m=1}^{\infty }\frac{{\left(\ell \right)}_{m}}{m{\left(k+\ell \right)}_{m}}\left\{\psi (k+\ell +m)-\psi (k+m)\right\}, \end{array}$$

which, when combined with (80), readily yields (71). □

Let k≥1 be fixed, and assume that *X* is uniformly distributed on (0,1). Set $T_{n}:=T_{n}\left(k,1,1\right)$ to be as in (5).

**Proposition** **4.** *Under the assumptions above, we have*

(83) $$\begin{array}{c} n^{1/2}\left\{T_{n}\left(k\right)-\log k+\psi \left(k\right)\right\}\overset{d}{\to }N\left(0,\psi^{\prime }\left(k\right)+1\right). \end{array}$$

**Proof.**  Denote by $\left\{\omega_{n}:n\geq 1\right\}$ an i.i.d. sequence of exponentially distributed E(1) r.v.’s, with mean 1. For each n≥0, set

$$S_{n}:=\sum_{i=1}^{n}\omega_{i}\overset{d}{=}\Gamma \left(n\right),$$

and set, in view of Lemma 1, for each 1≤i≤k≤n+2,

$$\Delta_{i,n}^{(k)*}:=\frac{S_{i+k-1}-S_{i-1}}{S_{n+1}}\overset{d}{=}U_{k,n}\overset{d}{=}\beta \left(k,n-k+1\right).$$

We keep in mind that $\left\{\Delta_{i,n}^{(k)*}:0\leq i\leq n-k+2\right\}\overset{d}{=}\left\{\Delta_{i,n}^{\left(k\right)}:0\leq i\leq n-k+2\right\}$ and that $\left\{\Delta_{i,n}^{(k)*}:0\leq i\leq n-k+2\right\}$ is independent of $S_{n+1}\overset{d}{=}\Gamma \left(n+1\right)$. Set further

(84) $$\begin{array}{ccc} T_{n}^{*}\left(k\right) & := & \sum_{i=1}^{n-k+1}\left\{-\log\left(\frac{1}{k}S_{n+1}\Delta_{i,n}^{(k)*}\right)\right\}\\ & = & \sum_{i=1}^{n-k+1}\left\{-\log\left(\frac{1}{k}\left(S_{i+k-1}-S_{i-1}\right)\right)\right\}\\ & = & \sum_{i=1}^{n-k+1}\left\{-\log\left(S_{i+k-1}-S_{i-1}\right)+\log k\right\},\; \end{array}$$

and

(85) $$\begin{array}{ccc} \;\;T_{n}^{**}\left(k\right) & := & \left(n-k\right)\left\{-\log n-\left(-\log S_{n+1}\right)\right\}. \end{array}$$

Set, likewise,

(86) $$\begin{array}{ccc} & & T_{n}^{*}\left(k\right):=\sum_{i=1}^{n-k+1}\left\{-\log\left(\frac{n}{k}\Delta_{i,n}^{(k)*}\right)\right\}\\ & = & \sum_{i=1}^{n-k+1}\left\{-\log\left(S_{i+k-1}-S_{i-1}\right)+\log k-\log n-\left(-\log S_{n+1}\right)\right\}\\ & = & T_{n}^{*}\left(k\right)+T_{n}^{**}\left(k\right). \end{array}$$

Observe that $T_{n}^{*}\left(k\right)\overset{d}{=}T_{n}\left(k\right)$. Moreover, the r.v.’s $T_{n}^{*}\left(k\right)$ and $T_{n}^{**}\left(k\right)$ are independent. Note further that, for each 0≤i≤n−k+2, $S_{i+k-1}-S_{i-1}\overset{d}{=}\Gamma \left(k\right)$ and $S_{n+1}\overset{d}{=}\Gamma \left(n+1\right)$. By (53), it follows that, for each 0≤i≤n−k+2,

$$E\left(-\log\left(S_{i+k-1}-S_{i-1}\right)\right)=-\psi \left(k\right)\;\mathrm{and}\;E\left(-\log S_{n+1}\right)=-\psi \left(n+1\right),$$

whence

$$\begin{array}{ccc} E\left(T_{n}^{*}\left(k\right)\right) & = & \left(n-k\right)\left\{-\psi (k)+\log k\right\} \end{array}$$

and

$$\begin{array}{ccc} E\left(T_{n}^{**}\left(k\right)\right) & = & \left(n-k\right)\left\{-\log n+\psi (n+1)\right\}. \end{array}$$

We have, therefore,

$$\begin{array}{ccc} E\left(T_{n}^{*}\left(k\right)\right) & = & E\left(T_{n}^{*}\left(k\right)\right)+E\left(T_{n}^{**}\left(k\right)\right)\\ & = & \left(n-k\right)\left\{-\psi (k)+\log k+\psi (n+1)-\log n\right\}. \end{array}$$

Next, we note that the $R^{2}$-valued r.v.’s $\{(-\log\left(S_{i+k-1}-S_{i-1}\right),S_{i}-S_{i-1}:i\geq 1\}$ form a stationary *k*-dependent sequence. Since, by (53) and (56), for all i≥1,

$$\mathrm{Var}\left(-\log\left(S_{i+k-1}-S_{i-1}\right)\right)=\psi^{\prime }\left(k\right)\;\mathrm{and}\;\mathrm{Var}\left(S_{i}-S_{i-1}\right)=1,$$

the partial sums of this sequence are asymptotically normal in $R^{2}$. It follows readily that, as n→∞,

(87) $$\begin{array}{ccc} & & n^{-1/2}\left(T_{n}^{*}\left(k\right)-E\left\{T_{n}^{*}\left(k\right)\right\},T_{n}^{**}\left(k\right)-E\left\{T_{n}^{**}\left(k\right)\right\}\right)\\ & = & n^{-1/2}\sum_{i=1}^{n-k+1}\{-\log\left(S_{i+k-1}-S_{i-1}\right)+\psi \left(k\right)-\log k\\ & & -\log n+\psi \left(n+1\right)-\left(-\log S_{n+1}\right)\}\\ & & \;\overset{d}{\to }\;N\left(\left[\begin{array}{c} 0\\ 0 \end{array}\right],\left[\begin{array}{cc} \psi^{\prime }\left(k\right) & 0\\ 0 & 1 \end{array}\right]\right). \end{array}$$

Here, we have made use of the fact that, as n→∞,

$$\psi \left(n+1\right)-\log n=\frac{1+o(1)}{2n},$$

so that, in (88),

$$n^{-1/2}\psi \left(n+1\right)\to 0.$$

Likewise, making use of (70), we see that

$$\begin{array}{ccc} & & \mathrm{Cov}\left(T_{n}^{*}\left(k\right),T_{n}^{**}\left(k\right)\right)=\sum_{i=1}^{n-k+1}\mathrm{Cov}\left(-\log\left(S_{i+k-1}-S_{i-1}\right),-\log S_{n+1}\right)\\ & = & \left(n-k\right)\mathrm{Cov}\left(-\log S_{k},-\log S_{n+1}\right)=\left(n-k\right)\psi^{\prime }\left(n+1\right)\to 1\;\mathrm{as}\;n\to \infty , \end{array}$$

Making use of (69), an easy argument shows that, in turn,

(88) $$\begin{array}{ccc} & & n^{-1/2}\sum_{i=1}^{n-k+1}\{-\log\left(S_{i+k-1}-S_{i-1}\right)+\psi \left(k\right)-\log k\\ & & -\log n-\left(-\log S_{n+1}\right)\}\;\overset{d}{\to }\;N\left(0,1+\psi^{\prime }\left(k\right)\right). \end{array}$$

In view of (87), we readily obtain (83) from this last relation. □

**Remark** **3.** *Let G(u):=inf{x:F(x)≥u} for 0<u<1 denote the quantile function of X. Assume that both F(·) and G(·) are continuous. In this case, we may define the quantile density function of X by g(u)=1/f(G(u)), which is continuous for 0<u<1. We may then set, for 1≤i≤n,*

$$\begin{array}{ccc} X_{i+k-1,n}-X_{i-1,n} & = & G\left(U_{i+k-1,n}\right)-G\left(U_{i-1,n}\right)\\ & = & \frac{1+o_{P}\left(1\right)}{f(G(i/n))}\left\{U_{i+k-1,n}-U_{i-1,n}\right\}. \end{array}$$

*Having proved Theorem 1 for f(x)=1, the conclusion for a general f follows by routine arguments based on this observation, relating uniform spacings to general spacings. We omit the details.*

## Data Availability

The original contributions presented in this study are included in the article. Further inquiries can be directed to the corresponding author.

## References

[1] Darling D.A. (1953). On a class of problems related to the random division of an interval. Ann. Math. Stat..

[2] Blumenthal S. (1968). Logarithms of sample spacings. SIAM J. Appl. Math..

[3] Deheuvels P., Derzko G. (2003). Exact laws for products of uniform spacings. Austrian J. Stat..

[4] Hájek J., Šidák Z. (1967). Theory of Rank Tests.

[5] Pyke R. (1965). Spacings. J. R. Stat. Soc. B.

[6] Pyke R. (1972). Spacings revisited. Proceedings of the 6th Berkeley Symposium.

[7] Deheuvels P. Spacings and applications. Proceedings of the 4th Pannonian Symposium on Mathematical Statistics.

[8] Cressie N. (1976). On the logarithms of high-order spacings. Biometrika.

[9] Cressie N. (1978). Power results for tests based on high-order gaps. Biometrika.

[10] Shao Y., Hahn M.G. (1995). Limit theorems for the logarithm of sample spacings. Stat. Probab. Lett..

[11] Deheuvels P., Derzko G. (2005). Tests of fit based on products of spacings. Probability, Statistics and Modelling in Public Health.

[12] del Pino G. (1975). On the asymptotic distribution of some goodness of fit tests based on spacings. Bull. Inst. Math. Stat..

[13] del Pino G. (1979). On the asymptotic distribution of *k*-spacings with application to goodness-of-fit tests. Ann. Stat..

[14] Czekała F. (1996). Normalizing constants for a statistic based on logarithm of disjoint *m*-spacings. Appl. Math..

[15] Spanier J., Oldham K.B. (1987). An Atlas of Functions.

[16] Gradshteyn I.S., Ryzhik I.M. (1965). Tables of Integrals, Series and Products.

[17] Abramowitz M., Stegun I.A. (1970). Handbook of Mathematical Functions.

[18] Johnson N.J., Kotz S., Balakrishnan N. (1994). Continuous Univariate Distributions, Volume 1.

[19] Johnson N.J., Kotz S., Balakrishnan N. (1995). Continuous Univariate Distributions, Volume 2.

[20] David H.A. (1981). Order Statistics.

[21] Sukhatme P.V. (1937). Tests of significance for samples of the *χ*^2^ population with two degrees of freedom. Ann. Eugen..

[22] Malmquist S. (1950). On a property of order statistics from a rectangular distribution. Skand. Aktuarietidskr..

[23] Csörgő M., Révész P. (1981). Strong Approximations in Probability and Statistics.

